# Proteomic Profiling Comparing the Effects of Different Heat Treatments on Camel (*Camelus dromedarius*) Milk Whey Proteins

**DOI:** 10.3390/ijms18040721

**Published:** 2017-03-28

**Authors:** Hicham Benabdelkamel, Afshan Masood, Ibrahim O. Alanazi, Dunia A. Alzahrani, Deema K. Alrabiah, Sami A. AlYahya, Assim A. Alfadda

**Affiliations:** 1 Proteomics Resource Unit, Obesity Research Center, College of Medicine, King Saud University, P.O. Box 2925(98), Riyadh 11461, Saudi Arabia; afsmasood@ksu.edu.sa (A.M.); dalzahrani@kacst.edu.sa (D.A.A.); dalrabiah@kacst.edu.sa (D.K.A.); aalfadda@ksu.edu.sa (A.A.A.); 2 The National Center For Genomic Technology (NCGT), Life Science and Environment Research Institute, King Abdulaziz City for Science and Technology (KACST), P.O. Box 6086, Riyadh 11461, Saudi Arabia; ialenazi@kacst.edu.sa; 3 National Center for Biotechnology, Life Science and Environment Research Institute, King Abdulaziz City for Science and Technology (KACST), P.O. Box 6086, Riyadh 11461, Saudi Arabia; salyahya@kacst.edu.sa; 4 Department of Medicine, College of Medicine, King Saud University, P.O. Box 2925(38), Riyadh 11461, Saudi Arabia

**Keywords:** camel milk, whey protein, heat treatment, 2D-DIGE, proteomics, matrix-assisted laser desorption/ionization-Time of Flight (MALDI-TOF)

## Abstract

Camel milk is consumed in the Middle East because of its high nutritional value. Traditional heating methods and the duration of heating affect the protein content and nutritional quality of the milk. We examined the denaturation of whey proteins in camel milk by assessing the effects of temperature on the whey protein profile at room temperature (RT), moderate heating at 63 °C, and at 98 °C, for 1 h. The qualitative and quantitative variations in the whey proteins before and after heat treatments were determined using quantitative 2D-difference in gel electrophoresis (DIGE)-mass spectrometry. Qualitative gel image analysis revealed a similar spot distribution between samples at RT and those heated at 63 °C, while the spot distribution between RT and samples heated at 98 °C differed. One hundred sixteen protein spots were determined to be significantly different (*p* < 0.05 and a fold change of ≥1.2) between the non-heated and heated milk samples. Eighty protein spots were decreased in common in both the heat-treated samples and an additional 25 spots were further decreased in the 98 °C sample. The proteins with decreased abundance included serum albumin, lactadherin, fibrinogen β and γ chain, lactotransferrin, active receptor type-2A, arginase-1, glutathione peroxidase-1 and, thiopurine S, etc. Eight protein spots were increased in common to both the samples when compared to RT and included α-lactalbumin, a glycosylation-dependent cell adhesion molecule. Whey proteins present in camel milk were less affected by heating at 63 °C than at 98 °C. This experimental study showed that denaturation increased significantly as the temperature increased from 63 to 98 °C.

## 1. Introduction

The one-humped camel (*Camelus dromedarius*) lives in the arid and semi-arid desert regions of the Middle East and produces a considerable amount of milk despite this hot and dry environment. It is thus a valuable source of nutrition, providing both milk and meat in these areas of the world [1,2].

Camel milk is popular locally and is mainly consumed as fresh raw milk or as soured milk [1,2]. Its characteristics and composition are attributed to many factors such as the age and breed of the camel, health of the animal, feeding conditions, stage of lactation, calving number, season, and geographical location; the latter two are the most important factors [1,3,4]. High amounts of active compounds, critical for the nutritional requirements of human neonates and adults, have been identified in camel milk [5].

Camel milk is mainly composed of lipids, proteins, lactose (as the major oligosaccharide), ash or minerals, numerous vitamins, essential amino acids, nucleotides, and other metabolites [6,7]. An extensive review by Konuspayeva (1993–2006) provides the mean values of the components as: fat matter 3.78 ± 1.31, total protein 3.19 ± 0.60, dry matter 11.98 ± 1.78, lactose 4.34 ± 0.54, and ash 0.81 ± 0.08 [8]. More recently, it was also reported to possess various health and therapeutic benefits, including antibacterial, immunological, anti-carcinogenic, anti-diabetic, and anti-hypertensive properties. Because of its low allergenic properties, camel milk is recommended for consumption for neonates and children allergic to bovine milk [1].

The protein in the milk is classified into three broad classes: colloidal casein, soluble whey, and milk fat globule membrane proteins (MFGMP) [9,10]. Proteins are highly heat labile and easily undergo conformational changes with increasing temperatures [11]. Heat treatment of milk is an essential step, performed domestically and industrially, to render milk safe for human consumption and improve its shelf life [12]. Heat treatment methods include thermization, extended pasteurization at low temperature, rapid pasteurization at high temperature, and sterilization at an ultra-high temperature [13,14]; pasteurization is the method of choice. However, in many rural areas, people consume milk after boiling it using charcoal or gas [15]. This practice can cause inefficient killing of microorganisms or deterioration in the quality of milk when excessive heat is applied.

As thermal treatment is the major step involved in the processing of milk and milk products, its effects on total milk and on whey proteins has been extensively studied. Several immune-active compounds identified in bovine, camel, caprine, and human milk are reduced after pasteurization [16,17,18,19].

Thus far only a few studies have investigated the effect of heat treatment on camel milk whey protein and a comprehensive proteome of camel milk and of proteins secreted in the lactating mammary gland of camels is not yet available [10]. There are limited studies that have examined the sameusing proteomic approach. Felfoul et al. have recently used a semi-quantitative-based one dimensional-gel electrophoresis (1D-GEL) and LC-MS/MS approach, at 80 °C for 1 h to study these changes [20].

We have thus carried out this study to determine quantitatively the changes in dromedary camel whey protein with relation to temperature using two different heating strategies, namely heating at 63 and 98 °C in comparison to room temperature, in order to identify proteins affected or stable by heating using a complementary proteomics and 2D-DIGE mass spectrometry approach.

## 2. Results and Discussion

The effects of heat on the whey proteins present in camel milk were examined using two-dimensional differential gel electrophoresis and matrix-assisted laser desorption ionization-time of flight mass spectrometry (2D-DIGE-MALDI/TOF). We used quantitative proteomics to obtain an overall representation of the changes in the profile of acid whey proteins present in camel milk after heating. Earlier studies on camel milk employed a targeted approach, studying a fraction, or a specific protein; no comprehensive profiling is currently available for the entire proteome. In this study, we found that a number of whey proteins were differentially affected by heating, with distinct changes noted at the different selected temperatures.

### 2.1. Overall Changes in Whey Proteins, Caused by Heating, Shown by 2D-DIGE

**Figure 1:**
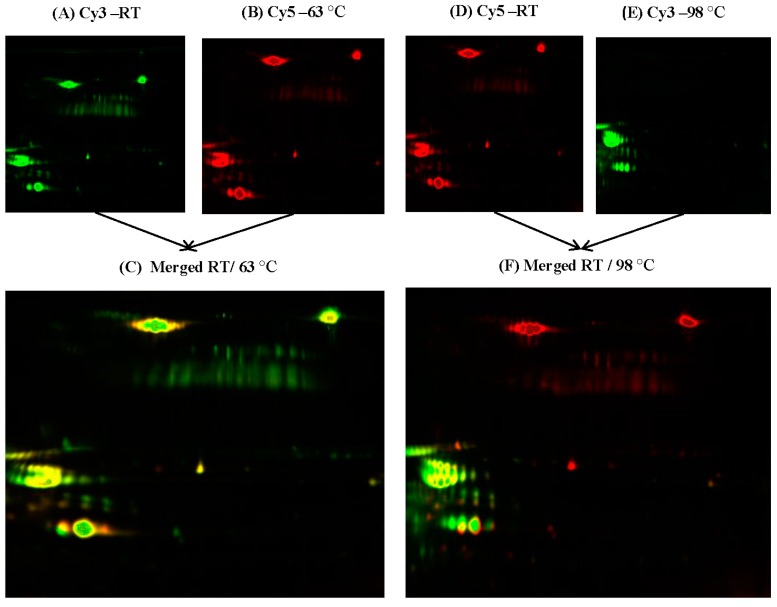
Two-dimensional fluorescence difference gel electrophoresis (2D-DIGE) image showing heat-treated and non-heat-treated samples of camel milk. Whey proteins were focused on linear IPG (immobilized pH gradient) strips (pH 3–11, 24 cm) and then separated using 12.5% polyacrylamide gels. Individual 2D-DIGE gel images of camel milk samples: (**A**) Cy3 and (**D**) Cy5 represent non-heated samples (at room temperature); gel images (**B**) Cy5 and (**E**) Cy3 represent samples heated at 63 and 98 °C, respectively; images (**C**,**F**) represent a channel overlap image between Cy3 and Cy5.

The 2D-DIGE experiments were carried out in triplicate and yielded reproducible spot patterns for all the milk samples, i.e., those at—RT, 63 and 98 °C. Approximately 1300 spots were mapped to the gels. The protein spots for the samples at room temperature (red/green), those at 63 °C (red), and those at 98 °C (green) were compared (Figure 1A,B,D,E). The gel images showed a high degree of protein similarity between the RT and those heated at 63 °C, as shown by the number of yellow spots (Figure 1C); protein similarity was minimal between the samples maintained at room temperature and those heated at 98 °C (Figure 1F); yellow spots represent proteins with the same isoelectric point, molecular weight, and nearly equal protein fluorescence intensity. Statistical analysis of the gels was conducted using the Progenesis statistical software v.3.3 (Nonlinear Dynamics, Newcastle, UK). One hundred and ninety protein spots, exhibiting a fold change of ≥1.2, and ANOVA *p*-value ≤ 0.05, were chosen for identification via mass spectrometry.

### 2.2. PCA Analysis

**Figure 2:**
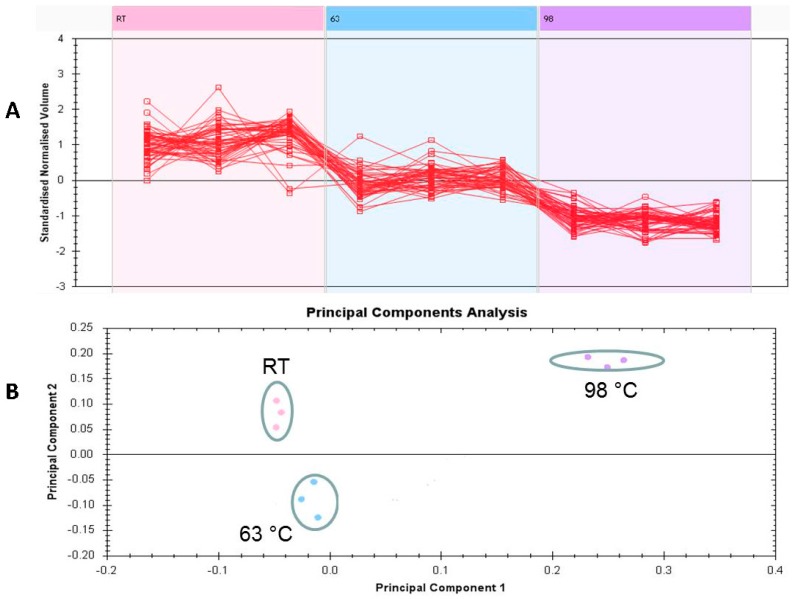
(**A**) Expression profiles, separated into clusters of expression patterns, indicating the number of spots for each cluster. Each line represents the standardized abundance of a spot across all gels and belongs to one of the clusters generated by hierarchical cluster analysis (Progenesis Same Spots); and (**B**) PCA plot of the first two principal components showing 85.15% variability of the selected spots. Colored dots and numbers represent gels and spots, respectively.

The unsupervised principal component analysis (PCA) bi-plot of gels and spots (Figure 2B) shows distinct gel grouping that agrees with the experimental groups. PCA plot of the two first principal components explained 85.15% of the selected spot’s variability within the three groups. The differentially abundant spots showed expression pattern clusters according to their abundant patterns based on a hierarchical clustering analysis (Figure 2A). The clustering pattern showed minimum amount of change in the protein intensities for the selected spots between RT and 63 °C while the difference for the same spots between RT and 98 °C was higher and significantly different.

### 2.3. MALDI-TOF-MS

The 190 protein spots of interest were excised from the preparative 2-D gels. After trypsin digestion, the digested spots were analyzed by MALDI-TOF-MS; then, 116 protein spots, isolated using MALDI-TOF-MS analysis, were successfully identified using the MASCOT Swiss Prot database. The database search results, with the coverage and score, are listed in Table S1. Thirty-one proteins were successfully matched with high certainty with entries in the *C. dromedarius* database, and an additional 85 assignments were made by matching to known homologous peptides identified in other mammalian databases (Table 1). Not all spots of interest could be identified because some proteins were low in abundance and did not yield sufficiently intense mass of fingerprints; other spots were mixtures of multiple proteins.

### 2.4. Changes in Abundance of Proteins after Application of Different Heating Strategies

Heat treatment of milk subjects it to the highly complex Maillard reaction, which greatly affects the structure and properties of its constituents including the whey proteins [21]. The reaction commonly occurs between the milk sugar, lactose, and the lysine residues of the milk proteins, leading to formation of large high molecular weight aggregates. This reaction is mostly seen to take place between the different casein fractions and lactoglobulin. β-Lactoglobulin is present in the milk of other dairy animals, such as the cow and the buffalo, but is characteristically absent from camel milk; this renders the composition of camel milk similar to that of human milk and accounts for the decreased allergenic property of camel milk. Camel milk is also known to have a lower amount of lactose in comparison to bovine milk. The major whey proteins in camel milk are α-lactoglobulin, lactoferrin, lactoperoxidase, serum albumin, immunoglobulin G, and secretory immunoglobulin A. Thermal denaturing, and aggregation or gelation, in the bovine milk has been extensively studied, while only a few studies have examined it in camel milk [16,22]. We found that the major fraction of proteins affected by heat treatment included 61% enzymes, 20% binding proteins, 10% cell adhesion proteins, 5% proteins involved in the immune response, 2% transport proteins, and 2% others.

### 2.5. Proteins That Showed Decreased Abundance after Heating at 63 or 98 °C, Compared to Room Temperature

We found that a total of 80 protein spots, corresponding to samples heated at 63 and 98 °C, were decreased, with an appreciable difference in fold change. Statistical analysis revealed a significant decrease in the levels of these spots, which related to proteins including: lactotransferrin, spot #348 (fold change of −1.417/−8.383 at 63/98 °C, respectively); lactadherin, spot #564 (fold change of −2.652/−14.107 at 63/98 °C, respectively); serum albumin, spot #333 (fold change of −1.466/−4.597 at 63/98 °C, respectively); cytochrome P450 11B2 mitochondrial, spot # 360 (fold change of −1.243/−9.594 at 63/98 °C respectively); arginase-1, spot #553 (fold change of −2.488/−2.541 at 63/98 °C, respectively); heat shock 70 kDa protein 14, spot #587 (fold change of −1.365/−16.099 at 63/98 °C, respectively); succinate dehydrogenase cytochrome b560 subunit, spot #597 (fold change of −2.043/−15.508 at 63/98 °C, respectively); Ig α-1 chain C region #462 (fold change of −1.117/−34.941 at 63/98 °C, respectively) (Table 1). Intensive heat treatment affected the functional properties [15] and solubility of milk serum proteins [23,24]. Caseins and whey proteins are engaged in protein aggregates, found in heat-treated milk; the formation of intermolecular disulfide bonds is mostly responsible for this heat-induced protein association. Proteins, especially enzymes, are heat-sensitive, and are denatured at higher temperatures. Therefore, moderate and high heating temperatures eradicated 68% of the protein spots in our dataset, in which enzymes accounted for 61% of the spots. Spots relating to mitochondrial enzymes, such as cytochrome P450 11B2 mitochondrial spot and succinate dehydrogenase cytochrome b560, were completely absent at 98 °C. Spots relating to lactoferrin, a protein involved in iron metabolism and having antioxidant activity, were found to have variable intensities, this was likely caused by the different temperatures, because some of the spots representing the isoforms of lactoferrin were absent at 63 °C, while others were present.

### 2.6. Proteins Stable at 63 °C with Decrease at 98 °C Compared to RT

Camel milk whey has been shown to be more heat stable than bovine or buffalo whey. A number of studies reported that the denaturation of camel milk was reported to be lower (32%–35%) than that reported for bovine whey proteins (70%–75%) at 80 °C for 30 min [16,25]. We identified 25 protein spots stable and showed no significant change when heated at 63 °C; the same proteins were found to be significantly decreased, with a greater fold change, when heated at 98 °C (Table 1). This group of proteins included apolipoprotein A-I, spot #256 (fold change of 1.003/−2.337 at 63/98 °C, respectively); carboxypeptidase N catalytic chain, spot # 803 (fold change of 1.213/−4.831 at 63/98 °C, respectively); fibrinogen γ chain, spot #539 (fold change of 1.140/−1.760 at 63/98 °C, respectively); glutathione peroxidase 1, spot #409 (fold change of 1.231/−13.938 at 63/98 °C, respectively); haptoglobin, spot #615 (fold change of 1.011/−3.377 at 63/98 °C, respectively); Ig α-1 chain C region, spot #462 (fold change of 1.117/−34 at 63/98 °C, respectively); lactotransferrin, spot #500 (fold change of 1.156/−7.593 at 63/98 °C, respectively), peptidoglycan recognition protein 1, spot #396 (fold change of 1.044/−26.553 at 63/98 °C respectively); plasminogen, spot #616 (fold change of 1.008/3.657 at 63/98 °C, respectively); steroid 17-α-hydroxylase/17,20 lyase, spot #644 (fold change of 1.079/−10.522 at 63/98 °C, respectively); and succinate dehydrogenase cytochrome b560 subunit, spot #539 (fold change of 1.140/−1.760 at 63/98 °C, respectively). The heating of milk causes the denaturing of the globular whey protein, which can result in the exposure of reactive amino acid side groups, normally buried within the native conformation. The denaturation process is either reversible, where partial unfolding of the whey proteins with a loss of helical structure, or irreversible where an aggregation process occurs involving sulfhydryl (–SH)/disulfide (S–S) interchange reactions and other intermolecular interactions, such as hydrophobic and electrostatic interactions [26,27,28]. Protective proteins, and enzymes possessing antibacterial, antimicrobial, and antioxidant properties, such as lactoferrin, lactoperoxidase, and peptidoglycan recognition protein (PGRP), were destroyed after heating at 98 °C but remained unchanged after heating at 63 °C. PGRP, which possesses anti-carcinogenic properties, controls metastasis, stimulates the immune system, and exerts antimicrobial activity, is particularly important in this group of proteins [29].

### 2.7. Proteins That Increased in Abundance after Heating at 63 or 98 °C, Compared to Room Temperature

We found that eight protein spots had increase in abundance after heating at 63 and 98 °C; the fold change was greater at the latter temperature. These spots related to: α-lactalbumin, spot #1069 (fold change of 1.4/11.9 at 63/98 °C, respectively); copper chaperone for superoxide dismutase, spot #1041 (fold change of 1.5/1.6 at 63/98 °C, respectively); ERI1 exoribonuclease 2, spot #1167 (fold change of 1.2/15.6 at 63/98 °C, respectively); and glycosylation-dependent cell adhesion molecule 1, spot #985 (fold change of 1.3/3.9 at 63/98 °C, respectively). Using SDS-PAGE, Farah showed that the heat denaturation of the individual camel whey proteins is not as pronounced as that in bovine whey proteins; more intensive heat treatment of camel milk is necessary to obtain the same degree of denaturation as that observed in bovine milk [22]. Heat treatment at more than 63 °C results in unfolding of the globular structure of whey proteins and they denature [30,31]. These non-native structures can then form aggregates with other unfolded monomers or aggregate with other types of protein molecules [32,33], forming co-aggregates [34], which is a characteristic feature of the Maillard reaction.

### 2.8. Proteins with an Increase in Abundance at 63 °C but a Decrease at 98 °C and RT

We found that three protein spots, relating to hemopexin spot, #413 (fold change of 1.258/−16.162 at 63/98 °C, respectively); lactate dehydrogenase, spot #856 (fold change of 1.923/−1.445 at 63/98 °C, respectively); and ADP-ribosylation factor GTPase-activating, spot #796 (fold change of 1.280/−2.509 at 63/98 °C, respectively), were found to be differentially regulated between the two heating temperatures. These spots increased in abundance at 63 °C but decreased significantly, or were undetectable at, 98 °C. When subjected to different temperatures at low pH, milk forms different types of gels, causing the whey proteins to partially unfold and adopt an intermediate state, depending on the degree of heating [35]. Hattem et al. showed that the highest level of denaturation occurs after heating 90 °C for 30 min, and the lowest at 63 °C for 30 min; the rate of protein denaturation is proportional to increases in temperature [36]. Moderate thermal treatment (60–70 °C) induces structural unfolding in milk proteins, whereas at higher temperatures, protein aggregation occurs [37]. Lactate dehydrogenase is part of the glycolytic pathway mediating the oxidative/reductive connection between pyruvate and lactic acid. Avallone et al. found that lactate dehydrogenase is heat stable and retains most of its activity up to 70 °C; it, then, loses its activity upon further heating. This agrees with the presence of the spots indicating lactate dehydrogenase, where we observed a band at 63 °C, which was absent at 98 °C [38]. The enzyme levels in buffalo milk are used as markers for pasteurization because of their sensitivity to heat inactivation [39]. Hemopexin is a plasma glycoprotein present in mature camel milk; it acts as a binding protein for iron and possesses antioxidant activity. In their proteomic study, Le et al. used ion exchange fractionation to identify hemopexin in the colostrum and mature bovine milk, with a higher abundance in the colostrum. The presence of this protein in camel milk signifies the important properties of camel milk [40].

## 3. Materials and Methods

### 3.1. Animals and Sample Collection

Camel milk was obtained from three different healthy camels from Saudi Arabia (cream, tan, and black subtypes of *Camelus dromedarius*), fed annually by grazing, from a local farm in the city of Al Majmaah, Saudi Arabia. Approximately 600 mL of milk, from each subtype of camel, was collected at 5:00 p.m. by milking into sterile cans and transported to the ORC (Obesity Research Center), College of Medicine, King Saud University proteomic lab in an ice box at 4 °C within 4 h of collection. Upon reaching the laboratory, the pH of the milk samples was determined to be 6.6 with a pH meter (PB11, Sartorius, Gottingen, Germany).

### 3.2. Sample Preparation

**Figure 3:**
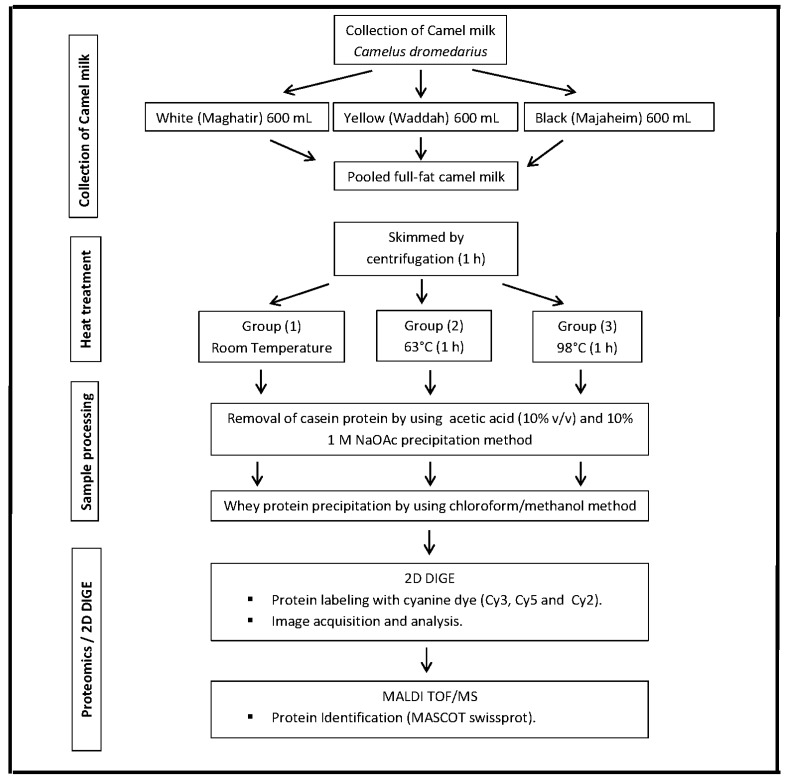
Schema of the proteomics workflow, showing the key experimental steps in this study.

The milk samples from the three different subtypes of the same breed of camel were pooled together to produce a homogenous sample. The pooled sample was then triplicated to represent three independent biological samples for the application of heat treatment. The initial step, conducted on the samples, involved the removal of fat by centrifugation at 400× *g* and 4 °C for 30 min to obtain skim milk (Figure 3).

### 3.3. Heat Treatment

From the previously skimmed milk samples, one group of the samples, kept at room temperature, served as the control. The other two groups served as thermally treated samples that were heated at 63 and 98 °C for 1 h each, using a thermostatically controlled water bath (Shaking Water Bath SHWB10, Cole Parmer, San Diego, CA, USA) with rapidly circulated water. The treated and untreated samples were again triplicated for assessing reproducibility.

### 3.4. Whey Protein Extraction

Following the individual heat treatments, casein was precipitated out, as described previously, by adding 10% acetic acid (10% *v*/*v*), followed by 10% 1 M NaOAc, with gentle shaking; the samples were allowed to rest for 30 min at 35 °C between each addition. After acidification, the samples were centrifuged at 20,000× *g* for 30 min at 5 °C and the supernatants were collected [41]. The acidified whey proteins were then precipitated out using the methanol/chloroform (4:4:1; *v*:*v*:*v*) precipitation method at room temperature, and centrifuged at 5000× *g* for 5 min to remove the upper phase. Another three volumes of methanol were added to the supernatant, and the samples were again mixed and centrifuged at 16,000× *g* for 5 min to obtain the protein pellets, which were dried at room temperature in a vacuum concentrator (Concentrator Plus, Eppendorf, Hamburg, Germany) [42].

### 3.5. Protein Labeling with Cyanine Dyes

The protein pellets were solubilized in a labeling buffer (7 M urea, 2 M thiourea, 30 mM Tris–HCl, 4% CHAPS, pH 8.5). The insoluble material was pelleted by centrifugation at 12,000× *g* at room temperature for 5 min. Protein concentrations were determined in triplicate using the 2D-Quantkit (GE Healthcare, Piscataway, NJ, USA), and the pH of the samples was adjusted to 8.5 using NaOH (100 mM). The proteins were labeled using 400 pmol CyDye™ DIGE Fluor dyes (GE Healthcare, Buckinghamshire, UK) in 1 μL of dimethylformamide (DMF) and then mixed with a sample containing 50 μg of protein. Samples were incubated on ice for 30 min in the dark. The labeling reaction was terminated by adding 1 μL of 10 mM lysine. Each sample was covalently labeled with a Cy3 or Cy5 fluorophore. A mixture of equal amounts of protein, isolated from each sample, was labeled with Cy2 and used as an internal standard (see Table S2).

### 3.6. Two-Dimensional Electrophoresis, Image Scanning, and Preparative Gel

First dimension analytical gel electrophoresis was performed as follows. Five Immobiline Dry Strips (24 cm, pH 3–11; GE Healthcare, Uppsala, Sweden) were passively re-hydrated at 30 V for 12 h. This was followed by isoelectric focusing using an Ettan IPGphor IEF unit (GE Healthcare, Sweden). Focusing was performed at 20 °C, at 50 μA per strip, according to the following hold sequence: (1) 500 V for 3 h, (2) 1000 V for 3 h, (3) 8000 V for 4 h, and (4) 8000 V for 6.25 h totalling up to 50,000 Vhrs. IPG (immobilized pH gradient) strips were then stored at −80 °C until second-dimension separation. Before the second-dimension separation, the IPG strips were first equilibrated with dithiothreitol for 15 min at room temperature with gentle stirring, then with 5 mM Tris–HCl (pH 8.8), 6 M urea, 30% glycerol, 2% SDS, and 65 mM DTT. The strips were then equilibrated for an additional 15 min in the same solution containing 250 mM iodoacetamide. Polyacrylamide fixed gels (12.5%) were prepared on low fluorescence glass using a 2-D Optimizer (NextGen Sciences, London, UK). Next, we performed second-dimension separation using sodium dodecyl sulfate polyacrylamide gel electrophoresis (SDS-PAGE; Ettan DALT six vertical units (GE Healthcare, Uppsala, Sweden), at 15 °C, 1 W per gel, for 1 h, and then at 2 W per gel until the bromophenol blue dye front reached the bottom of the gel. Then, the gels were scanned at the appropriate individual excitation and emission wavelengths using the Typhoon Trio Imager fluorescence gel scanner (GE Healthcare) with the values of filters and photomultiplier optimized for Cy3, Cy5, and Cy2.

### 3.7. Colloidal Coomassie Blue Staining of the Preparative Gel

Total protein (1 mg) was obtained from a pool of equal protein amounts of the nine camel milk samples. This was denatured in a lysis buffer, then mixed in a rehydration buffer. Gels were fixed in 40% (*v*/*v*) ethanol with 10% (*v*/*v*) acetic acid (overnight) and then washed (3×, 30 min each, ddH_2_O). The gels were incubated (1 h, 34% (*v*/*v*) methanol containing 17% (*w*/*v*) ammonium sulphate and 3% (*v*/*v*) phosphoric acid) prior to the addition of 0.5 g/L Coomassie G-250. After five days the stained gels were briefly rinsed with Milli-Q water and stored until the spots could be picked and identified by MS.

### 3.8. Protein Identification by MALDI-TOF MS

The spots from Coomassie-stained gels were excised manually, washed, and digested according to a previously published protocol [43]. The mixture of tryptic peptides (1 µL), derived from each protein, was spotted onto a MALDI (Matrix-assisted laser desorption/ionization) target (384 anchorchip MTP 800 µm Anchorchip; Bruker Daltonics, Bremen, Germany) together with 0.8 μL of matrix (10 mg α-cyano-4-hydroxycinnamic acid (CHCA) in 1 μL of 30% CH_3_CN and 0.1% aqueous CF_3_COOH) and then left to dry (RT) before MS analysis. Spectra were acquired using a MALDI-TOF MS (UltraFlexTrem, Bruker Daltonics, Bremen, Germany) in the positive mode with target voltage of 25 kV and pulsed ion extraction voltage of 20 kV. The reflector voltage was set to 21 kV and detector voltage to 17 kV. Peptide mass fingerprints (PMF) were calibrated against a standard (Peptide Calibration Standard II, Bruker Daltonics). The PMF were processed using the Flex AnalysisTM software (version 2.4, Bruker Daltonics). The MS data were interpreted using BioTools v3.2 (Bruker Daltonics), together with the MASCOT search algorithm (version 2.0.04 updated 09/05/2015; Matrix Science Ltd., London, UK). MASCOT search parameters were set as follows: fixed propionamide modification of cysteine, oxidation of methionine as variable modification, one missed cleavage site (such as in the case of incomplete trypsin hydrolysis), and a mass tolerance of 100 ppm. Identified proteins were accepted as correct if they showed a MASCOT score greater than 56 and *p* < 0.05. Not all spots of interest could be identified because some proteins were low in abundance and did not yield a sufficiently intense mass of fingerprints; other spots were mixtures of multiple proteins.

### 3.9. Image Acquisition

DIGE images were analyzed using the Progenesis Same Spots v3.3 software (Nonlinear Dynamics Ltd., Newcastle, UK). First, images were aligned. Then an automatic vector tool, using prominent spots, was employed to detect 400 total vectors for warping and aligning the gel images with a reference image of one internal standard across and within each gel. Gel groups were designated according to the experimental design; normalized spot volume was used to select statistically significant spots. The Progenesis Same Spots v3.3 software was used to calculate the normalized volume (NV) of each spot, on each gel, for Cy3 and Cy5, to Cy2 spot volume ratio. The software performs log transformations of the spot volumes to generate normally distributed data. Log normalized volume (LNV) was used to quantify differential expression. Independent direct comparisons were made between differently heat-treated milk samples. Fold differences and *p*-values were calculated using one-way ANOVA. All spots were pre-filtered and manually examined before applying the statistical criteria (ANOVA test, *p* ≤ 0.05 and fold ≥1.2). Instead of spot intensities, normalized spot volumes were used for statistical analysis. Only those spots that fulfilled the abovementioned statistical criteria were submitted for MS analysis.

## 4. Conclusions

This study aimed to identify the total protein composition during the heat treatment of camel milk whey proteins at a medium temperature of 63 °C and a high temperature of 98 °C for 60 min. A total of 116 protein were detected as significantly changing using 2-DIGE and identified by MALDI-MS. The obtained results showed that camel whey proteins were significantly affected by heat treatment at 98 °C and several proteins disappeared completely from the gel patterns; however, the whey proteins remain slightly stable under heat treatment at 63 °C for 60 min. This experimental study showed that denaturation increased significantly as the temperature increased from 63 to 98 °C. The fold change in the abundance of proteins identified between RT and 63 °C ranged from 15%–61% and for RT and 98 °C from 79%–98%. Further studies are needed to elucidate the mechanism involved in the heat denaturation of camel milk whey proteins and study the mechanisms in further detail.

## Figures and Tables

**Table 1:** Identified proteins, with changes in abundance, after application of heat treatment at 63 and 98 °C, compared with RT (room temperature). Table shows average ratio values for 63 °C/room temperature and 98 °C/room temperature, with their corresponding levels of fold changes and one-way ANOVA (*p*-value ≤ 0.05).

Spot Number	Accession Number	Protein Name	Function of Protein	ANOVA (*p*)	Fold Change			
					63 °C/RT	Expression	98 °C /RT	Expression
219	Q9TUM0	Lactotransferrin	Enzyme	0.001	−1.251	Down	−13.731	Down
242	P31428	Dipeptidase 1	Enzyme	0.005	−1.752	Down	−4.361	Down
247	Q9TUM0	Lactotransferrin	Enzyme	0.000	−1.505	Down	−5.241	Down
253	Q9TUM0	Lactotransferrin	Enzyme	0.000	−1.348	Down	−4.250	Down
255	P01009	α-1-Antitrypsin	Enzyme	0.001	−1.187	Down	−4.648	Down
256	P02647	Apolipoprotein A-I	Binding protein	0.001	1.003	Non-significant	−2.337	Down
270	P02675	Fibrinogen β chain	Enzyme	0.001	2.152	Down	−6.851	Down
276	Q8CG71	Prolyl 3-hydroxylase 2	Enzyme	0.030	−1.690	Down	−3.460	Down
280	P02679	Fibrinogen γ chain	Enzyme	0.003	−2.042	Down	−4.523	Down
285	P02679	Fibrinogen γ chain	Enzyme	0.004	−1.451	Down	−2.023	Down
288	Q32KP7	Uncharacterized protein C17orf64 homolog	Others	0.000	−1.264	Down	−2.313	Down
309	Q9TUM0	Lactotransferrin	Enzyme	0.000	−1.053	Down	−2.309	Down
316	Q9TUM0	Lactotransferrin	Enzyme	0.004	−1.187	Down	−1.709	Down
318	P79385	Lactadherin	Cell adhesion	0.000	−1.103	Down	−2.633	Down
333	P02768	Serum albumin	Enzyme	0.003	−1.466	Down	−4.597	Down
340	Q9TUM0	Lactotransferrin	Enzyme	0.001	−1.421	Down	−5.242	Down
338	Q9TUM0	Lactotransferrin	Enzyme	0.001	−1.421	Down	−5.242	Down
339	Q9TUM0	Lactotransferrin	Enzyme	0.013	−1.483	Down	−4.500	Down
343	Q9TUM0	Lactotransferrin	Enzyme	0.033	−1.512	Down	−4.958	Down
348	Q9TUM0	Lactotransferrin	Enzyme	0.014	−1.417	Down	−8.383	Down
353	Q9TUM0	Lactotransferrin	Enzyme	0.003	−1.297	Down	−10.836	Down
357	Q9TUM0	Lactotransferrin	Enzyme	0.001	−1.291	Down	−11.745	Down
358	Q9TUM0	Lactotransferrin	Enzyme	0.000	1.148	Non-significant	−22.961	Down
359	Q9TUM0	Lactotransferrin	Enzyme	0.000	1.031	Non-significant	−44.852	Down
360	P15539	Cytochrome P450 11B2, mitochondrial	Enzyme	0.004	−1.243	Down	−9.594	Down
361	Q6IML7	DnaJ homolog subfamily C member 27	Binding protein	0.007	−1.222	Down	−6.650	Down
368	Q9TUM0	Lactotransferrin	Enzyme	0.000	−1.060	Down	−67.105	Down
373	Q9TUM0	Lactotransferrin	Enzyme	0.000	1.042	Non-significant	−45.000	Down
384	P02750	Leucine-rich α-2-glycoprotein	Binding protein	0.001	−1.138	Down	−27.366	Down
396	Q9GK12	Peptidoglycan recognition protein 1	Binding protein	0.001	1.044	Non-significant	−26.553	Down
398	Q9TUM0	Lactotransferrin	Enzyme	0.000	1.095	Non-significant	−36.065	Down
400	Q7Z713	Ankyrin repeat domain-containing protein 37	Binding protein	0.007	1.295	Non-significant	−11.880	Down
407	Q9H3Z7	Protein ABHD16B	Binding protein	0.000	1.307	Non-significant	−15.143	Down
409	Q8MJ14	Glutathione peroxidase 1	Enzyme	0.000	1.231	Non-significant	−13.938	Down
413	P02790	Hemopexin	Transport protein	0.003	1.258	Up	−16.162	Down
433	Q9TUM0	Lactotransferrin	Enzyme	0.008	1.039	Non-significant	−6.116	Down
438	Q9TUM0	Lactotransferrin	Enzyme	0.000	1.115	Non-significant	−10.873	Down
440	Q8R0F8	Acylpyruvase FAHD1, mitochondrial	Enzyme	0.000	−1.419	Down	−1.708	Down
454	Q5E9H8	Probable G-protein coupled receptor 173	Enzyme	0.001	−1.213	Down	−29.425	Down
459	Q9TUM0	Lactotransferrin	Enzyme	0.002	−1.257	Down	−15.872	Down
462	P01876	Ig α-1 chain C region	Immune response protein	0.001	1.117	Non-significant	−34.941	Down
472	Q96SZ5	2-Aminoethanethiol dioxygenase	Enzyme	0.010	−1.358	Down	−2.038	Down
474	Q9TUM0	Lactotransferrin	Enzyme	0.002	−1.363	Down	−15.500	Down
492	Q95KI3	Endothelial cell-selective adhesion molecule	Cell adhesion	0.011	−1.374	Down	−3.065	Down
500	Q9TUM0	Lactotransferrin	Enzyme	0.000	1.156	Non-significant	−7.593	Down
501	P00435	Glutathione peroxidase 1	Enzyme	0.035	−1.218	Down	−3.432	Down
516	Q6GPH4	XIAP-associated factor 1	Binding protein	0.006	−1.068	Down	−5.456	Down
517	Q8NBT0	POC1 centriolar protein homolog A	Binding protein	0.023	−1.460	Down	−2.404	Down
539	P70097	Succinate dehydrogenase cytochrome b560 subunit, mitochondrial	Enzyme	0.002	1.140	Non-significant	−1.760	Down
287	P02679	Fibrinogen γ chain	Enzyme	0.003	−2.042	Down	−4.523	Down
543	Q68G74	LIM/homeobox protein Lhx8	Binding protein	0.009	−1.284	Down	−3.709	Down
540	Q68G74	LIM/homeobox protein Lhx8	Binding protein	0.009	−1.284	Down	−4.609	Down
546	Q6AYB4	Heat shock 70 kDa protein 14	Immune response protein	0.008	−1.993	Down	−2.279	Down
548	Q6AYB4	Heat shock 70 kDa protein 14	Immune response protein	0.007	−1.793	Down	−3.35	Down
553	Q2KJ64	Arginase-1	Enzyme	0.009	−2.488	Down	−2.541	Down
555	Q2KJ64	Arginase-1	Enzyme	0.087	−2.47	Down	−2.81	Down
561	P79385	Lactadherin	Cell adhesion	0.013	−1.910	Down	−6.104	Down
566	P79385	Lactadherin	Cell adhesion	0.002	−1.510	Down	−8.104	Down
562	P79385	Lactadherin	Cell adhesion	0.000	−1.480	Down	−11.000	Down
563	P79385	Lactadherin	Cell adhesion	0.001	−1.580	Down	−6.000	Down
564	P79385	Lactadherin	Cell adhesion	0.000	−2.652	Down	−14.107	Down
560	P79385	Lactadherin	Cell adhesion	0.000	−2.022	Down	−12.007	Down
568	P46065	Guanylyl cyclase-activating protein 1	Enzyme	0.000	−1.742	Down	−8.144	Down
570	P46065	Guanylyl cyclase-activating protein 1	Enzyme	0.000	−1.82	Down	−9.100	Down
573	P06323	T-cell receptor α chain V region CTL-F3	Immune response protein	0.05	−1.417	Down	−14.836	Down
576	P06323	T-cell receptor α chain V region CTL-F3	Immune response protein	0.002	−1.357	Down	−10.35	Down
572	P06323	T-cell receptor α chain V region CTL-F3	Immune response protein	0.004	−2.17	Down	−8.572	Down
574	P79385	Lactadherin	Cell adhesion	0.000	−2.304	Down	−18.431	Down
579	P79385	Lactadherin	Cell adhesion	0.000	−2.65	Down	−13.251	Down
578	Q9TUM0	Lactotransferrin	Enzyme	0.003	−1.357	Down	−17.075	Down
580	Q9TUM0	Lactotransferrin	Enzyme	0.000	−1.564	Down	−15.005	Down
582	P04217	α-1B-glycoprotein	Enzyme	0.002	−1.572	Down	−2.461	Down
584	P04217	α-1B-glycoprotein	Enzyme	0.015	−1.980	Down	−3.542	Down
585	Q9QYE2	Solute carrier organic anion transporter family member 1A6	Binding protein	0.000	1.036	Non-significant	−9.092	Down
586	Q9QYE2	Solute carrier organic anion transporter family member 1A6	Binding protein	0.003	1.259	Non-significant	−7.058	Down
587	Q6AYB4	Heat shock 70 kDa protein 14	Immune response protein	0.000	−1.365	Down	−16.099	Down
590	Q6AYB4	Heat shock 70 kDa protein 14	Immune response protein	0.000	−1.572	Down	−12.557	Down
588	Q28560	Activin receptor type-2A	Binding protein	0.007	−2.822	Down	−16.337	Down
592	Q28560	Activin receptor type-2A	Binding protein	0.000	−2.092	Down	−11.777	Down
594	Q2KJ64	Arginase-1	Enzyme	0.000	−1.917	Down	−14.343	Down
593	P34896	Serine hydroxymethyltransferase, cytosolic	Enzyme	0.000	−1.717	Down	−16.343	Down
595	Q13595	Transformer-2 protein homolog α	Binding protein	0.002	−1.295	Down	−8.503	Down
600	Q13595	Transformer-2 protein homolog α	Binding protein	0.01	−2.3	Down	−6.3	Down
596	P02675	Fibrinogen β chain	Enzyme	0.002	−1.154	Down	−7.207	Down
599	P02675	Fibrinogen β chain	Enzyme	0.005	−2.12	Down	−5.02	Down
597	P70097	Succinate dehydrogenase cytochrome b560 subunit, mitochondrial	Enzyme	0.000	−2.043	Down	−15.508	Down
604	Q68SB1	Double-stranded RNA-binding protein Staufen homolog 2	Binding protein	0.001	−1.002	Down	−1.651	Down
615	P00738	Haptoglobin	Transport protein	0.001	1.011	Non-significant	−3.377	Down
616	P00747	Plasminogen	Enzyme	0.001	1.008	Non-significant	−3.657	Down
621	P32261	Antithrombin-III	Enzyme	0.000	1.030	Non-significant	−5.271	Down
629	Q62803	Hyaluronidase PH-20	Enzyme	0.000	−1.025	Down	−8.901	Down
644	Q91Z85	Steroid 17-α-hydroxylase/17,20 lyase	Enzyme	0.000	1.079	Non-significant	−10.522	Down
662	P16261	Graves disease carrier protein/Mitochondrial solute carrier protein homolog	Enzyme	0.000	1.013	Non-significant	−14.205	Down
664	Q8HX86	Thiopurine *S*-methyltransferase	Enzyme	0.000	1.020	Non-significant	−15.146	Down
723	P10909	Clusterin	Binding protein	0.001	−1.132	Down	−2.792	Down
769	Q9NSQ0	Putative ribosomal RNA-processing protein 7 homolog B	Binding protein	0.000	−1.024	Down	−2.642	Down
793	O14791	Apolipoprotein L1	Binding protein	0.001	1.371	Non-significant	−2.684	Down
796	Q3MID3	ADP-ribosylation factor GTPase-activating protein 2	Binding protein	0.002	1.280	Up	−2.509	Down
803	P15169	Carboxypeptidase N catalytic chain	Enzyme	0.003	1.213	Non-significant	−4.831	Down
819	Q62803	Hyaluronidase PH-20	Enzyme	0.000	−1.264	Down	−3.213	Down
827	Q9UHR4	Brain-specific angiogenesis inhibitor 1-associated protein 2-like protein 1	Binding protein	0.000	−1.161	Down	−3.327	Down
828	Q9CTN5	Protein SIX6OS1	Binding protein	0.000	−1.327	Down	−4.083	Down
832	Q3UR70	Transforming growth factor-β receptor-associated protein 1	Binding protein	0.000	−1.417	Down	−3.559	Down
847	P24310	Cytochrome c oxidase subunit 7A1, mitochondrial	Enzyme	0.001	−1.507	Down	−3.142	Down
849	Q8NBP0	Tetratricopeptide repeat protein 13	Enzyme	0.000	−1.565	Down	−4.503	Down
856	Q29563	l-lactate dehydrogenase C chain	Enzyme	0.036	1.923	Up	−1.445	Down
917	P15522	Glycosylation-dependent cell adhesion molecule 1	Enzyme	0.000	1.249	Up	2.805	Up
985	P15522	Glycosylation-dependent cell adhesion molecule 1	Enzyme	0.000	1.283	Up	3.925	Up
1013	P15522	Glycosylation-dependent cell adhesion molecule 1	Enzyme	0.000	1.345	Up	2.301	Up
1041	O14618	Copper chaperone for superoxide dismutase	Others	0.060	1.535	Up	1.597	Up
1069	P00710	α-Lactalbumin	Enzyme	0.000	1.391	Up	11.925	Up
1090	P00710	α-Lactalbumin	Enzyme	0.001	1.321	Up	4.790	Up
1105	P00710	α-Lactalbumin	Enzyme	0.014	1.831	Up	2.547	Up
1147	Q8NFU3	Thiosulfate sulfurtransferase/rhodanese-like domain-containing protein 1	Enzyme	0.029	−1.661	Down	−2.326	Down
1155	Q86X95	Corepressor interacting with RBPJ 1	Enzyme	0.002	−1.389	Down	−3.541	Down
1167	A8K979	ERI1 exoribonuclease 2	Enzyme	0.000	1.196	Up	15.624	Up

## Supplementary Materials

Supplementary materials can be found at www.mdpi.com/1422-0067/18/4/721/s1.
