# Supplementary material for: Proteomic Profiling Comparing the Effects of Different Heat Treatments on Camel (Camelus dromedarius) Milk Whey Proteins
Source: Int J Mol Sci. 2017 Mar 28;18(4):721. doi: 10.3390/ijms18040721 (PMC5412307; doi:10.3390/ijms18040721)
Supplement: Supplementary file 1 [file ijms-18-00721-s001.pdf]

Supplementary Materials

Table S1. Mass spectrometry data showing significantly differentially abundant proteins identified in camel milk using 2D-DIGE [analysis type: matrix-assisted laser desorption/ionization; database: SwissProt; taxonomy: Mammalian].

Spot Number [a]	Accession Number [b]	Pi [c]	MW [d]	Cov% [e]	Score [f]
**219**	Q9TUM0	8.66	79158	21	59
**242**	P31428	5.9	46109	31	58
**247**	Q9TUM0	8.66	79158	25	62
**253**	Q9TUM0	8.66	79158	29	65
**255**	P01009	5.37	46878	36	68
**256**	P02647	5.56	30759	72	238
**270**	P02675	8.54	56577	55	205
**276**	Q8CG71	5.57	80959	19	63
**280**	P02679	5.37	52106	52	130
**285**	P02679	5.37	52106	52	130
**288**	Q32KP7	10.18	14535	38	65
**309**	Q9TUM0	8.66	79158	44	116
**316**	Q9TUM0	8.66	79158	48	184
**318**	P79385	7.61	53619	28	58
**340**	Q9TUM0	8.66	79158	35	114
**333**	P02768	5.92	71317	41	88
**338**	Q9TUM0	8.66	79158	46	150
**339**	Q9TUM0	8.66	79158	42	173
**343**	Q9TUM0	8.66	79158	38	131
**348**	Q9TUM0	8.66	79158	43	175
**353**	P02679	5.37	52106	46	145
**357**	Q9TUM0	8.66	79158	30	110
**358**	Q9TUM0	8.66	79158	44	143
**359**	Q9TUM0	8.66	79158	48	175
**360**	P15539	9.86	57449	18	60
**361**	Q6IML7	8.72	31329	33	64
**368**	Q9TUM0	8.66	79158	45	123
**373**	Q9TUM0	8.66	79158	50	171
**384**	P02750	6.45	38382	42	71
**396**	Q9GK12	9.1	21763	31	58
**398**	Q9TUM0	8.66	79158	36	128
**400**	Q7Z713	5.39	17650	52	60
**407**	Q9H3Z7	8.9	53205	16	57
**409**	Q8MJ14	6.74	22863	37	59

**413**	P02790	6.55	52385	48	96
**433**	Q9TUM0	8.66	79158	42	139
**438**	Q9TUM0	8.66	79158	48	158
**440**	Q8R0F8	7.59	25484	42	58
**454**	Q5E9H8	9.3	42307	25	60
**459**	Q9TUM0	8.66	79158	46	129
**462**	P01876	6.08	38486	42	58
**472**	Q96SZ5	5.68	30074	24	59
**474**	Q9TUM0	8.66	79158	48	146
**492**	Q95KI3	8.95	41206	22	58
**500**	Q9TUM0	8.66	79158	51	172
**501**	P00435	6.74	22930	40	60
**516**	Q6GPH4	8.57	35857	40	62
**517**	Q8NBT0	7.31	45551	28	60
**539**	P02675	5.37	52106	50	129
**543**	Q68G74	8.66	40445	21	73
**546**	Q6AYB4	5.41	55170	24	59
**553**	Q2KJ64	6.09	35272	34	62
**561**	P79385	6.15	46722	29	63
**562**	P79385	6.15	46722	25	58
**564**	P79385	6.15	46722	27	65
**568**	P46065	4.4	23723	62	64
**572**	P06323	9.13	14908	37	60
**579**	P79385	6.15	46722	34	75
**574**	P79385	6.15	46722	34	79
**578**	Q9TUM0	8.66	79158	25	92
**582**	P04217	5.58	54809	37	90
**585**	Q9QYE2	7.81	76169	19	57
**587**	Q6AYB4	5.41	55170	23	59
**588**	Q28560	5.62	58814	20	60
**594**	Q2KJ64	6.09	35272	36	61
**595**	Q13595	11.27	32726	39	58
**596**	P02675	5.37	52106	48	120
**539**	P70097	10.04	18714	50	65
**543**	Q68G74	8.66	40445	21	73
**548**	Q6AYB4	5.41	55170	24	55
**553**	Q2KJ64	6.09	35272	34	54
**561**	P79385	6.15	46722	29	63
**562**	P79385	6.15	46722	25	58
**560**	P79385	6.15	46722	27	65
**570**	P46065	4.4	23723	62	64
**576**	P06323	6.15	46722	34	75

**579**	P79385	6.15	46722	34	79
**578**	Q9TUM0	8.66	79158	25	92
**582**	P04217	5.58	54809	37	90
**586**	Q9QYE2	7.81	76169	19	59
**590**	Q6AYB4	5.41	55170	23	59
**588**	Q28560	5.62	58814	20	60
**593**	P34896	6.09	35272	36	61
**600**	Q13595	11.27	32726	39	58
**596**	P02675	5.37	52106	48	120
**597**	P70097	10.04	18714	50	65
**604**	Q68SB1	9.54	62870	30	57
**615**	P00738	6.13	45861	21	70
**616**	P00747	7.04	93247	30	77
**621**	P32261	6.1	52484	24	60
**629**	Q62803	6.09	59059	26	64
**644**	Q91Z85	6.32	58312	22	59
**662**	P16261	9.97	35375	30	61
**664**	Q8HX86	6.46	28752	35	59
**723**	P10909	5.89	53031	26	78
**769**	Q9NSQ0	9.73	12568	46	68
**793**	O14791	5.6	44009	32	59
**796**	Q3MID3	8.08	56863	26	85
**803**	P15169	6.86	52538	33	62
**819**	Q62803	6.09	59059	14	57
**827**	Q9UHR4	8.82	57189	19	58
**828**	Q9CTN5	5.45	67339	18	59
**832**	Q3UR70	6.24	98382	11	64
**847**	P24310	10.12	9169	60	70
**849**	Q8NBP0	6.56	97607	19	54
**856**	Q29563	7.62	36534	23	62
**917**	P15522	5.36	17281	30	59
**985**	P15522	5.36	17281	25	57
**1013**	P15522	5.36	17281	30	54
**1041**	O14618	5.32	29536	30	60
**1069**	P00710	5	14877	65	66
**1090**	P00710	5	14877	53	94
**1105**	P00710	5	14877	45	64
**1147**	Q8NFU3	5.85	12579	20	51
**1155**	Q86X95	5.82	81606	26	99
**1167**	A8K979	9.12	78549	22	66

[a] Spot numbers correspond to those included in the 2D-image.

[b] Protein accession number for SWISSPROT Database.

[c] Theoretical isoelectric point.

[d] Theoretical relative mass.

[e] % of peptide sequence Coverage.

[f] MASCOT score.

Table S2. Experimental design: nine samples of camel milk were run on five analytical 2D-DIGE gels; the samples were labeled randomly with Cy3 and Cy5; a pooled sample was used as an internal standard and was stained with Cy2.

GEL	CY3	CY5	CY2
**1**	**CM-RT (1)**	**CM-63°C (1)**	**POOL**
**2**	**CM-98°C (1)**	**CM-RT (2)**	**POOL**
**3**	**CM-63°C (2)**	**CM-98°C (2)**	**POOL**
**4**	**CM-RT (3)**	**CM-63°C (3)**	**POOL**
**5**	**CM-98°C (3)**	**-**	**POOL**
